# Aortic valve replacement in pediatric patients: 30 years single center experience

**DOI:** 10.1186/s13019-021-01636-2

**Published:** 2021-09-08

**Authors:** Johanna Schlein, Paul Simon, Gregor Wollenek, Eva Base, Günther Laufer, Daniel Zimpfer

**Affiliations:** 1 University Clinic of Cardiac Surgery, Medical University of Vienna, Waehringer Guertel 18-20, 1090 Vienna, Austria; 2 Department of Anaesthesia, Intensive Care Medicine and Pain Medicine, Division of Cardiac Thoracic Vascular Anaesthesia and Intensive Care Medicine, Medical University of Vienna, Vienna, Austria

**Keywords:** Congenital aortic valve disease, Pediatric aortic valve replacement, Pediatric mechanical aortic valve replacement, Pediatric homograft aortic valve replacement, Pediatric bioprosthetic aortic valve replacement

## Abstract

**Background:**

The choice of aortic valve replacement needs to be decided in an interdisciplinary approach and together with the patients and their families regarding the need for re-operation and risks accompanying anticoagulation. We report long-term outcomes after different AVR options.

**Methods:**

A chart review of patients aged < 18 years at time of surgery, who had undergone AVR from May 1985 until April 2020 was conducted. Contraindications for Ross procedure, which is performed since 1991 at the center were reviewed in the observed non-Ross AVR cohort. The study endpoints were compared between the mechanical AVR and the biological AVR cohort.

**Results:**

From May 1985 to April 2020 fifty-five patients received sixty AVRs: 33 mechanical AVRs and 27 biological AVRs. In over half of the fifty-three AVRs performed after 1991 (58.5%; 31/53) a contraindication for Ross procedure was present. Early mortality was 5% (3/60). All early deaths occurred in patients aged < 1 year at time of surgery. Two late deaths occurred and survival was 94.5% ± 3.1% at 10 years and 86.4% ± 6.2% at 30 years. Freedom from aortic valve re-operation was higher (*p* < 0.001) in the mechanical AVR than in the biological AVR cohort with 95.2% ± 4.6% and 33.6% ± 13.4% freedom from re-operation at 10 years respectively.

**Conclusions:**

Re-operation was less frequent in the mechanical AVR cohort than in the biological AVR cohort. For mechanical AVR, the risk for thromboembolic and bleeding events was considerable with a composite linearized event rate per valve-year of 3.2%.

## Background

Despite the encouraging results with aortic valve reconstruction, aortic valve replacement (AVR) might be required in pediatric patients with significant valve destruction after failed-repairs or interventions [1, 2]. Mechanical prostheses are available in small sizes (16 and 18 mm) and suitable for older children, but not for infants or small children. Annular enlargement techniques (Nicks procedure [3], Manougian procedure [4], Konno procedure [5]) can enable implantation of a larger prosthesis [6, 7]. The need for life-long anticoagulation accompanying the choice of a mechanical prosthesis can be challenging in the pediatric cohort due to the lack of compliance with medication and activity restraints [7]. Anticoagulation regimen needs special consideration in female patients regarding a later pregnancy. The use of biological valve replacement is complicated by accelerated structural valve deterioration, which is faster than that seen in adults because of a greater immune competency and an increased calcium metabolism in young patients [8]. Aortic homografts offer an option for patients, who need more complex reconstruction of the aortic root and serves small children and infants. In recent years decellularized homografts were introduced showing promising results, also in pediatric patients [9, 10].

We reviewed the contraindications for Ross procedure in the observed non-Ross AVR cohort and report on outcomes after mechanical, bio-prosthetic and homograft AVR in pediatric patients.

## Methods

### Patients

This single-center study was conducted at a tertiary center with a pediatric heart center consisting of specialized pediatric cardiac surgeons, anesthetists and cardiologists. The study was approved by the local ethics committee board and requirement for individual patient consent was waived. A chart review of all AVR surgeries performed in patients aged < 18 years at time of surgery from May 1985 until April 2020 was conducted. The biological AVR group consisted of aortic homografts or prosthetic bioprostheses. The choice of implanted prosthesis in infant patients were homografts. Mechanical valves were used when patient age and expected compliance rendered hypocoagulation possible. All patients in the mechanical AVR cohort were treated with phenprocoumon (goal INR 2.0–3.0). Overall compliance was good, in two (6.1%; 2/33) teenaged patients temporary discontinuation of anticoagulation and permanently prescribed medication was reported. In the observed biological AVRs lifelong antiplatelet therapy was pursued with acetylsalicylic acid. Antithrombotic management differed over the study period. In the more recent years patients were additionally discharged with temporary anticoagulation therapy (phenprocoumon, goal INR 2.0–3.0), which was discontinued after the first three postoperative months.

### Definitions

**Fig. 1:**
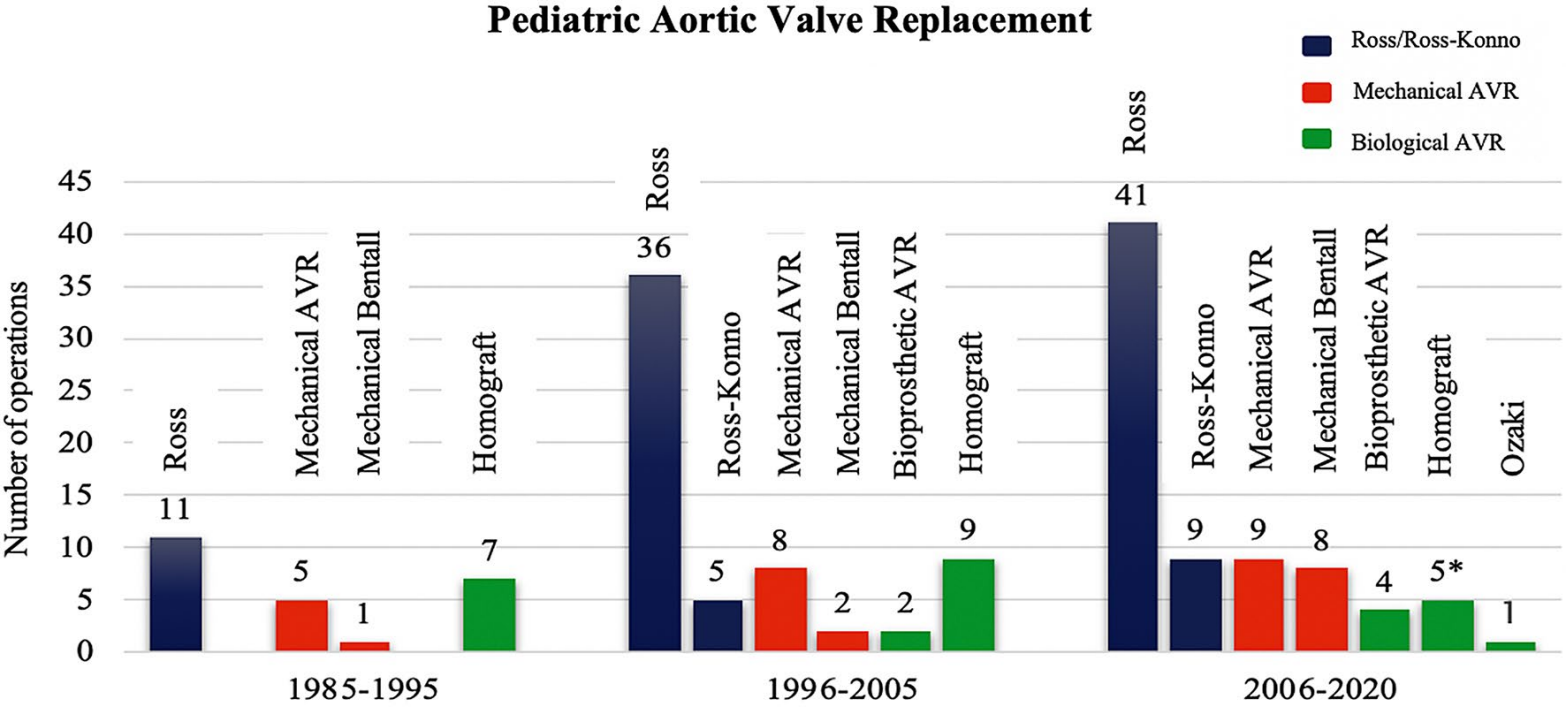
Frequency of AVR from 1985 to 2020. The frequency of AVR from May 1985 until April 2020 including the Ross procedure and Ozaki neo-cuspidization. Ross procedure was performed since 1991. ^*^Decellularized homografts. AVR, aortic valve replacement

Parameters were obtained and measured as described in the Guidelines for Reporting Mortality and Morbidity after Cardiac Valve Interventions [11]. Primary outcome parameters were survival and incidence as well as timing of re-operations. Early mortality was defined as death occurring with 30 days of surgery or prior to hospital discharge. Mortality was cross-checked with the national health insurance database. Survival status on April 30^th^, 2020 is known for 92.7% (51/55) of patients. Four patients were transferred for surgery from foreign centers and could not be followed-up in the database. Survival time for these patients was calculated until the last confirmed living follow-up. Patients were included with all their aortic valve replacements performed when aged < 18 years at the center. Three patients were included with two AVRs and one patient with three AVRs during the study period. Valve numbers are used in the tables. Two patients underwent left ventricular assist device (LVAD) implantation in the setting of subacute myocarditis 5 days after AVR and cardiomyopathy 3.6 years after AVR respectively. These patients were censored from further valve-related analysis at time of LVAD implantation. As a high-volume Ross center, we reviewed the contraindications for Ross procedure in the observed non-Ross AVR cohort. The Ross procedure is offered to pediatric patients at our center since 1991. Until April 2020 one-hundred-and-two pediatric patients underwent a Ross procedure. The frequency of AVR from 1985–2020 is seen in Fig. 1.

### Statistical analysis

Normally distributed continuous data was expressed as mean ± standard deviation, whilst skewed continuous data was expressed as median with interquartile range (IQR), and minimum and maximum. In order to identify significant differences between two subgroups, continuous variables were compared using the independent-samples Mann–Whitney U test and categorical variables with Fisher exact test. Time-related endpoints were analyzed and plotted using Kaplan–Meier actuarial survival curves accompanied by 95% confidence intervals. Freedom from re-intervention was compared in subgroups using the log-rank analysis. Patients, who did not experience outcome events were censored at the time of last follow-up. Univariable Cox-proportional hazard modelling was used to determine risk factors for mortality and re-operation. Linearized event rates per valve-year were calculated. Statistical significance was set at *p* < 0.05. Data was analyzed using the software package SPSS® 26 (IBM Corp., Chicago, Illinois, USA).

## Results

### Demographic and operative characteristics

From May 1985 until April 2020 60 AVRs were performed in 55 patients aged < 18 years at time of AVR. Patient and operative characteristics are given in Table 1. The patient cohort was predominantly male (70.9%; 39/55) and the median age at time of surgery was 12.1 years (IQR 7–15.4 years). Operative variables and the used valve types are given in Table 2 for the mechanical AVR (55%; 33/60: 22 mechanical AVR, 11 mechanical Bentall) and the biological AVR (45%; 27/60: 21 homograft AVRs, 6 bioprosthetic AVRs) cohorts respectively. The patients receiving the mechanical AVRs were older (*p* = 0.004) at time of surgery. In accordance the used valve sizes were smaller (*p* = 0.003) in the biological cohort. Median valve sizes and performed annular enlargement strategies are seen in Table 2. In over half of the fifty-three AVRs performed after 1991 (58.5%; 31/53) a contraindication for Ross procedure was present as seen in the Table 3.

**Table 1:** Baseline characteristics

Characteristic	Patients
Patient cohort	
Number	55
Male	39 (70.9)
Native aortic valve anatomy	
Unicuspidal	1 (1.8)
Bicuspid	19 (34.5)
Tricuspid	15 (27.3)
Quadricuspid	1 (1.8)
Unknown	19 (34.5)
Underlying diagnosis	
Isolated aortic valve lesion	39 (70.9)
Complex congenital heart disease	13 (23.6)
Diagnoses	
Shunt (VSD, ASD, PFO, PDA)	8 (14.5)
Aortic isthmus stenosis	9 (16.4)
Hypoplastic aortic arch	2 (3.6)
Tetralogy of Fallot	1 (1.8)
Double outlet right ventricle	2 (3.6)
Dextro-transposition of the great arteries	5 (9.1)
Congenitally corrected transposition of the great arteries	2 (3.6)
Endocardial fibroelastosis	7 (12.7)

**Operative**	**Valve implants**
Valve implants of cohort	
Number	60
Age (ywars) at time of surgery	
Neonates	2 (3.3)
< 1 (including neonates)	4 (6.7)
1–5	8 (13.3)
6–13	26 (43.3)
14–18	22 (36.7)
Aortic valve at replacement	
Native	45 (75)
Tirone David	2 (3.3)
Mechanical Bentall AVR	1 (1.7)
Bioprosthetic AVR	1 (1.7)
Homograft AVR	4 (6.7)
Neoaortic valve (ASO, left-ventricle-neo-aortic-valve-tunnel)	7 (11.6)

Values are presented as n, n (%)

ASD, atrial septum defect; ASO, arterial switch operation; AVR, aortic valve replacement; PDA, persistent ductus arteriosus; PFO, patent foramen ovale; VSD, ventricular septum defect

**Table 2:** Operative characteristics

Characteristic	Mechanical AVR	Homograft/bioprosthetic AVR	*p* value
Aortic valve replacement			
Mechanical AVR	22	–	
Mechanical Bentall	11	–	
Bioprosthetic AVR	–	6	
Homograft AVR	–	21	
Valves implanted^a^			
Mechanical/Bentall			
Carbomedics	12 (36.4)	–	
St. Jude Medical	11 (33.3)	–	
On–X	4 (12.1)	–	
Duromedics	3 (9.1)	–	
ATS	2 (6.1)	–	
Björk-Shiley Monostrut	1 (3)	–	
Bioprosthetic			
Inspiris Resilia	–	2 (7.4)	
Mosaic	–	2 (7.4)	
Sorin Pericarbon Stentless	–	1 (3.7)	
Tissuemed Freestyle Root	–	1 (3.7)	
Homograft			
Homograft bank	–	15 (55.6)	
Decellularized Corlife	–	5 (18.5)	
CryoLife homograft	–	1 (3.7)	
Age at time of surgery (years)	13.6 (9.7–15.7)	8.9 (2.4–14.4)	0.004
Weight at time of surgery (kg)	41.7 (24.5–63)	31.5 (12.5–50.2)	0.081
Height at time of surgery (cm)	149 (129–171)	139 (95–158)	0.064
BSA_Haycock_ at time of surgery	1.31 (0.93–1.77)	1.11 (0.56–1.53)	0.105
Median Valve size (mm)	21 (range 17–27)	20 (range 9–25)	0.003
Indication for surgery			0.740
Aortic valve stenosis	1 (3.0)	2 (7.4)	
Aortic valve regurgitation	23 (69.7)	17 (63)	
Mixed aortic valve lesion	9 (27.3)	8 (29.6)	
Bacterial endocarditis	2 (6.1)	2 (7.4)	> 0.99
Rheumatic valve disease	3 (9.1)	0 (0)	0.245
Prior cardiac surgery	23 (69.7)	17 (63)	0.596
Prior aortic valve surgery	12 (36.4)	14 (51.9)	0.297
Prior balloon aortic valvuloplasty	3 (9.1)	7 (25.9)	0.097
Time from last surgical aortic valve operation (years)	5 (1.6–7.9)	0.6 (0.04–3.3)	0.006
*Operative variable*			
ACCT (min)	95 (66.5–151)	95 (70–129)	0.969
CPB (min)	158 (103–233)	154 (90–235)	0.682
Circulatory arrest	5 (15.2)	1 (3.7)	0.209
Concomitant procedure	16 (48.5)	12 (44.4)	0.799
Aortic annulus enlargement			0.745
Nicks	1 (3)	0 (0)	
Manougian	2 (6.1)	0 (0)	
Konno	1 (3)	1 (3.7)	

Values are presented as n, n (%), median (interquartile range) or median (range minimum–maximum) in case of valve size (mm). Continuous variables were compared using the independent-samples Mann–Whitney *U* test and categorical variables with Fisher exact test

ACCT, Aortic cross clamp time; AVR, aortic valve replacement; BSA, body surface area; ccTGA, congenitally corrected transposition of the great arteries; CPB, cardiopulmonary bypass; VSD, ventricular septum defect

^a^*Carbomedics*; Sorin Spa, Milan, Italy; *St. Jude Medical*; St. Jude Medical Inc, St. Paul, Minn; *On-X*; On-X Life Technologies Inc, Austin, Tex; *Duromedics*; Edwards Lifesciences, Irvine Ca; *ATS*; ATS Medical Inc, Minneapolis, Minn; *Björk-Shiley Monostrut*; Pfizer Inc, New York, NY; *Inspiris Resilia*; Edwards Lifesciences, Irvine Ca; *Mosaic*; Medtronic plc, Dublin, Ireland; *Sorin Pericarbon Stentless*; Sorin Spa, Milan, Italy; *Tissuemed Freestyle Root;* Tissuemed, Leeds, England; Decellularized *Corlife*; Corlife, Hannover, Germany; *CryoLife*; CryoLife, Kennesaw, GA

**Table 3:** Contraindications for Ross procedure

Contraindication	n (%)
Any contraindication, including complex congenital heart disease	31 (100)
Patient’s parents were against a Ross procedure	3 (9.7)
Bicuspid pulmonary valve	6 (19.4)
Tricuspid, but dysplastic or insufficient pulmonary valve	3 (9.7)
Size discrepancy between the aortic and the pulmonary valve at time of surgery	2 (6.5)
Massive adhesions between the aortic and the pulmonary root	1 (3.2)
Coronary anatomy	2 (6.5)
Connective tissue disease	3 (9.7)
Marfanoid habitus with hyperextensibility of the joints and a dilated pulmonary artery	1 (3.2)
Suitable pulmonary homograft not available at time of surgery (1999)	1 (3.2)
Due to hematoma of the aorta ascendens at the cannulation site a mechanical Bentall was performed	1 (3.2)

Values are presented as n, n (%)

### Early outcomes

Postoperative outcomes are listed in Table 4. There were three early deaths (11.1%, 3/27) due to multi organ failure in the biological AVR cohort and no early deaths (0/33) in the mechanical AVR cohort. The procedural early mortality rate for the AVR cohort is 5% (3/60). All early deaths occurred in patients aged < 1 year at time of surgery. Deaths are summarized in Table 5.

**Table 4:** Postoperative outcomes

Characteristic	Mechanical AVR	Homograft/bioprosthetic AVR	*p* value
Permanent pacemaker insertion	2 (6.1)	0 (0)	0.497
Reoperation for bleeding	1 (3)	0 (0)	> 0.99
Reoperation for mitral regurgitation	1 (3)	1 (3.7)	> 0.99
Coronary ischemia	1 (3)	0 (0)	> 0.99
Ventilation (days)^a^	1 (0–2.5)	1 (0–1)	0.398
ICU stay (days)^a^	3.5 (1.3–5)	2 (1.5–3)	0.080
Hospital stay (days)^a^	18 (12.5–21.5)	11 (8–14)	0.001
Peritoneal dialysis	0 (0)	2 (7.4)^b^	0.198
ECMO	0 (0)	3 (11.1)^c^	0.085
Early mortality	0 (0)	3 (11.1)	0.085

Values are presented as n, n (%), median (interquartile range). Continuous variables were compared using the independent-samples Mann–Whitney *U* test and categorical variables with Fisher exact test

AVR, aortic valve replacement; ECMO, extracorporeal membrane oxygenation; ICU, intensive care unit

^a^Times of the three patients, who died and one LVAD-receiving patient are excluded

^b^In one case peritoneal dialysis had already been instated prior AVR

^c^In one case ECMO had already been implanted prior AVR

**Table 5:** Early and late deaths

Valve No. (sex)	Surgery (year)	Age	Diagnosis	Previous interventions	Concomitant procedure	Death (postoperative days/years)	Cause of death
*Early deaths*							
No. 29 (m)	Homograft (2002)	19 days	Critical aortic stenosis with endocardial fibroelastosis, MV stenosis, admitted to the center in moribund state on the 8th day of life after home birth	Surgical valvulotomy	LVOT enlargement Konno, MV-reconstruction, ECMO	First postoperative day	Cardiorespiratory failure in MOV
No. 12 (m)	Homograft (1995)	17 days	Critical aortic stenosis with endocardial fibroelastosis, MV regurgitation	Surgical valvulotomy	MV-reconstruction, ECMO	7 days	Cardiorespiratory failure in MOV
No. 25 (f)	Freestyle Pulmonary Root (2000)	3 months	Critical aortic stenosis with endocardial fibroelastosis, subvalvular aortic stenosis, MV stenosis, Shone’s with borderline left ventricle structures	Surgical valvulotomy	LVOT enlargement Konno	28 days Mitral valve replacement 27 days after initial AVR	Cardiorespiratory failure in MOV
*Late deaths*							
No. 18 (m)	Homograft (1998)	14.9 years	Aortic regurgitation (tricuspid) VSD	VSD closure Aortic valve repair		16.5 years AVR re-operation (6.8 years)	Myocardial infarction (posterior wall)
No. 24 (f)	Homograft (2000)	13.8 years	Combined aortic lesion (bicuspid), ascendens ectasia, coarctation, Turner syndrome Endocarditis (staphylococcus aureus) with valve dehiscence in pseudo aneurysm of the aortic root after mechanical Bentall not performed at center	Coarctation repair Mechanical Bentall		18.8 years	Unknown aetiology

AVR, aortic valve replacement; ECMO, extracorporeal membrane oxygenation; LVOT, left ventricular outflow tract; MOV, multi organ failure; MV, mitral valve; VSD, ventricular septal defect

### Follow-up

#### Patient-related follow-up

Two late deaths occurred and survival was 94.5% ± 3.1% at 10 years, 86.4% ± 6.2% at 20 years and at 30 years (Fig. 1). One patient died from myocardial infarction 16.5 years after AVR and one patient from unknown causes 18.8 years after AVR.

#### Valve-related follow-up

**Fig. 2:**
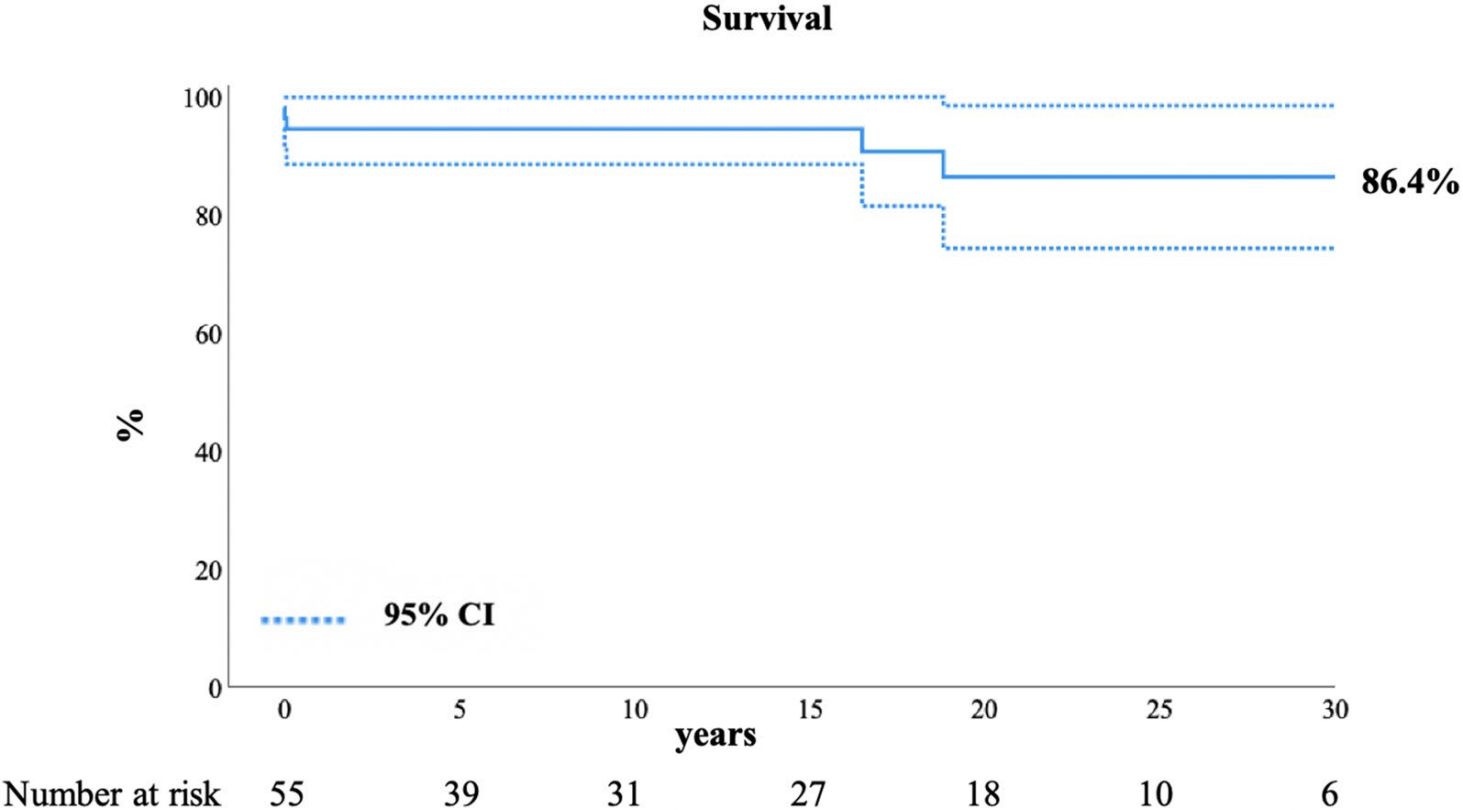
Survival following AVR. Kaplan–Meier estimated overall survival with 95% confidence interval (CI). AVR, aortic valve replacement

**Fig. 3:**
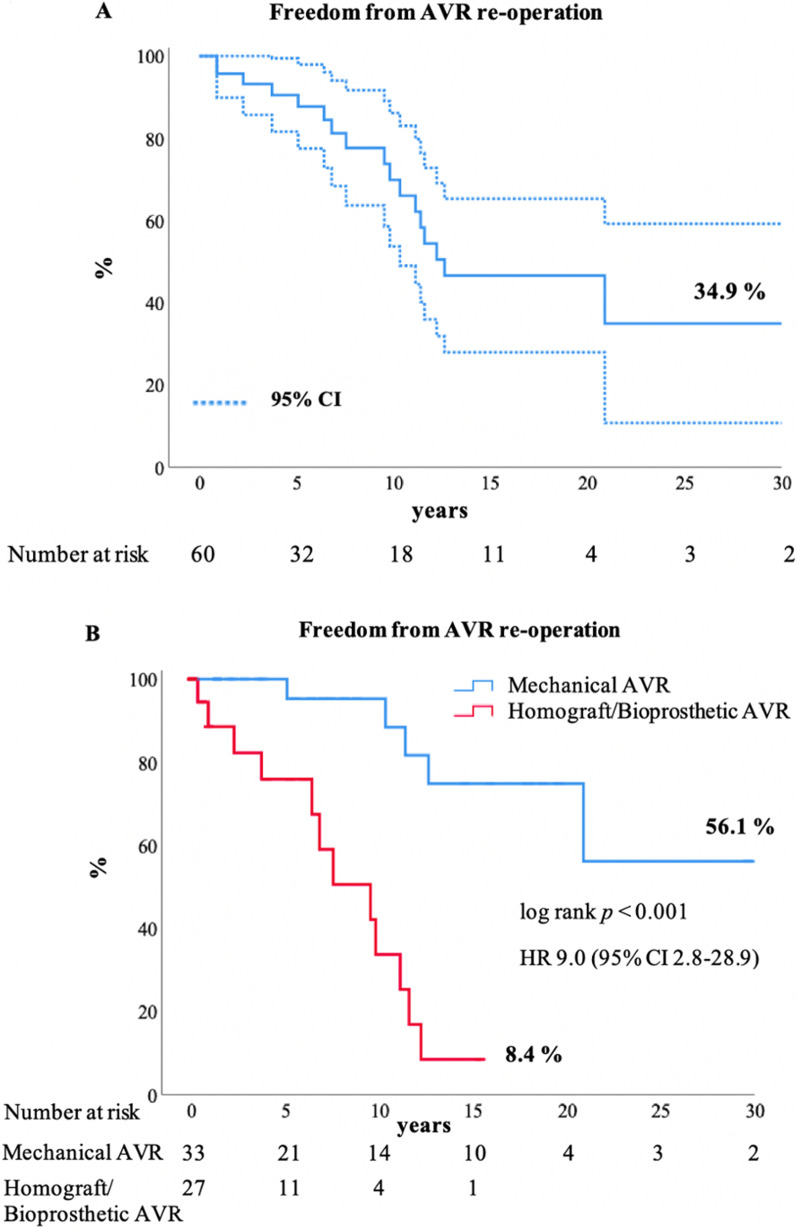
Freedom from aortic valve re-operation. **A** Kaplan–Meier estimated freedom from aortic valve re-operation. Curve with 95% confidence interval (CI). **B** Freedom from aortic valve re-operation in patients with mechanical AVR and biological AVR. Kaplan–Meier estimated freedom from aortic valve re-operation in patients following mechanical AVR versus patients following biological (homograft, bioprosthetic) AVR. AVR, aortic valve replacement

As shown in Fig. 2, freedom from aortic valve re-operation was 69.9% ± 8.3% at 10 years, 46.6% ± 9.5% at 20 years and 34.9% ± 12.4% at 30 years. Freedom from aortic valve re-operation was significantly higher (*p* < 0.001) in the mechanical AVR compared to the biological AVR cohort with 95.2% ± 4.6% and 33.6% ± 13.4% freedom from reoperation at 10 years respectively (Fig. 3). At univariable Cox proportional hazard analysis smaller implanted valve size was a risk factor for re-operation (HR 0.8 for each increase in valve size mm; *p* = 0.019). Five mechanical AVRs (15.2%; 5/33) and 12 biological AVRs (44.4%; 12/27: 11 homograft AVRs and one bioprosthetic valve) were re-operated at a median of 11.4 years (IQR 7.7–16.8 years) after mechanical AVR and at a median of 7.2 years (IQR 2.6–10.8 years) after biological AVR (*p* = 0.082). The valve replacements requiring re-operation are detailed in Table 6.

**Table 6:** Valve replacements requiring re-operation

Valve No. (sex)	Surgery (year)	Age at time of surgery (years)	Valve size (mm)	Reason for aortic valve replacement re-operation	Time from initial to aortic valve replacement re-operation (years)		Performed re-operation (Valve No.^a^)
*Mechanical AVR*							
No. 2 (m)	Saint Jude Medical (1986)	15.4	23	Paravalvular leak		5.1	Mechanical AVR
No. 3 (m)	Duromedics (1986)	15.5	19	Paravalvular leak		20.9	Mechanical AVR
No. 26 (f)	Carbomedics Bentall (2001)	7.8	23	Stenosis and regurgitation due to circular pannus formation in LVOT		11.4	Mechanical Bentall
No. 31 (m)	Carbomedics-TopHat (2003)	9.2	23	Pannus formation		10.3	Mechanical AVR
No. 32 (f)	Carbomedics-TopHat (2003)	6.1	19	Patient-prosthesis mismatch		12.6	Mechanical AVR
*Biological AVR*							
No. 6 (f)	Homograft (1989)	15.4	21	Regurgitation		0.3	Mechanical AVR (No. 7)
No. 8 (f)	Homograft (1992)	2.4	20	Regurgitation		7.6	Ross
No. 11 (m)	Homograft (1994)	10.7	19	Mixed aortic valve disease		3.7	Homograft (No. 15)
No. 13 (m)	Homograft (1995)	14.8	21	Endocarditis		11.1	Ross
No. 14 (f)	Homograft (1997)	17.9	22	Extensive structural valve degeneration due to calcification was noticed during aortic arch surgery		9.5	Mechanical AVR
No. 15 (m)	Homograft (1998)	14.4	22	Regurgitation		6.4	Mechanical AVR
No. 16 (m)	Homograft (1998)	6	21	Mixed aortic valve disease		9.8	Ross
No. 17 (m)	Homograft (1998)	0.2	10	Regurgitation		0.9	Homograft (No. 20)
No. 18 (m)	Homograft (1998)	14.9	21	Endocarditis		6.8	Homograft
No. 19 (m)	Sorin Pericarbon Stentless (1999)	8.9	21	Stenosis		2.3	Mechanical AVR (No. 28)
No. 20 (m)	Homograft (1999)	1.1	16	Regurgitation		12.2	Mechanical Bentall (No. 44)
No. 30 (f)	Homograft (2003)	7.8	20	Endocarditis		11.6	Biological Bentall

AVR, aortic valve replacement; LVOT, left ventricular outflow tract

^a^Valve No. is specified if reoperation valve was included in the study as AVR surgery had been performed when patient was aged < 18 years (Three patients received two included valves: No. 6 and No. 7, No. 11 and No. 15, No. 19 and No. 28; one patient received three included valves: No. 17, No. 20 and No. 44)

There were three bleeding events in the mechanical AVR cohort (1.2% per valve-year). One 17-year-old female patient had to undergo laparoscopic surgery for corpus rubrum bleeding in the setting of over anticoagulation (initial international normalized ratio (INR) at admission: 9.9, subsequent INR controls at admission day were not measurable). The other two patients were in goal INR range at time of event. For mechanical AVR the linearized event rate per valve-year was 0.41% for valve thrombosis, and 1.6% for embolism (two transient ischemic attacks in one patient, two strokes in one patient). One patient with a Saint Jude Medical valve underwent emergency surgery for valve thrombosis with cardiogenic shock 41 days after initial AVR. In the setting of reduced left ventricular function and with valve opening being sufficient after intraoperative debridement of thrombotic material and rinsing with alteplase, the valve was not explanted. In the biological AVR cohort no bleeding or thromboembolic events occurred. The linearized event rate per valve-year for endocarditis was 6.5% in the biological AVR cohort. No endocarditis occurred in the mechanical AVR cohort, but pannus formation (0.8% per valve-year) and paravalvular leak (0.8% per valve year) occurred.

## Discussion

The choice of AVR remains challenging in the pediatric cohort regarding hemodynamic profile and limitation of valve durability. Preoperative counseling for patients and their families is indispensable for taking each patient’s individual social characteristics and needs into consideration.

A valve preserving strategy is aimed for to postpone AVR until older age and therefore somatic growth resulting in a likely decreased periprocedural risk and depending on deference of AVR the option of an adult size valve. In our cohort 55% (33/60) of patients had undergone at least one aortic valve intervention (surgical or percutaneous) prior to AVR and the median time from the last surgical aortic valve intervention was shorter (*p* = 0.006) in the biological AVR cohort than in the mechanical AVR cohort with 0.6 years (IQR 0.04–3.3 years) and 5 years (IQR 1.6–7.9 years) respectively, as patients in the mechanical AVR cohort were older (*p* = 0.004) at time of surgery and therefore suitable for mechanical AVR with greater implanted valve sizes (*p* = 0.003) than the patients in the biological cohort.

Two patients (3.3%; 2/60) underwent surgery during neonatal period and 4 patients (6.6%; 4/60; including the two neonates) were younger than < 1 year at time of surgery. AVR in neonates and infants might become necessary as salvage surgery, when more conservative surgical approaches or percutaneous interventions have been unsuccessful in establishing an acceptable hemodynamic situation. These patients represent a high-risk group with increased periprocedural complications and mortality rates. Woods et al., reported an in-hospital mortality after aortic valve replacement (Ross-Konno, Ross, homograft AVR) in neonates and infants of 18% (29/160) with 28% (12/43) for neonates and 14% (17/117) for infants. Of the three AVR groups, those who underwent homograft AVR had the highest mortality rate (40%; 6/15; all infants) [12]. In a meta-analysis Etnel et al. reported a pooled early mortality of 7.3% for mechanical AVR and 12.8% for homograft AVR [13].

Freedom from aortic valve re-operation was significantly higher (*p* < 0.001) in the mechanical AVR than in the biological AVR cohort with 95.2% and 33.6% freedom from reoperation at 10 years respectively. Re-operation was seen in the mechanical cohort due to patient-prosthesis mismatch (20%; 1/5), pannus formation (40%; 2/5) and paravalvular leak (40%; 2/5). In the pediatric cohort younger patients will experience patients-prosthesis mismatch, when outgrowing the implanted prosthesis regardless of mechanical or biological AVR. Re-operation for paravalvular leak might become necessary also late after initial implantation. Khan et al. report a higher (*p* < 0.001) 5-year freedom from composite endpoint of re-intervention and death of 95% for mechanical AVR (n = 36) than for homograft AVR (n = 74) with 52% [14]. Etnel et al. calculated a pooled event rate for aortic valve re-operation of 1.1% per year for mechanical AVR compared to 5.4% per year for homograft AVR [13]. Consistent with other studies [7, 14, 15] biological AVR has a higher re-operation rate than mechanical AVR. However, the risk for bleeding or thromboembolic events is not to be neglected in the mechanical AVR cohort with an estimated pooled event rate of 0.76% per year for thromboembolism and valve thrombosis, and 0.39% per year for bleeding [13].

Decellularized aortic homografts might offer an additional AVR option for pediatric patients. Horke et al., who compared the pediatric data from the ARISE Registry for aortic decellularized homografts with the results of recent meta-analyses for pediatric AVR [13, 16], show that AVR with decellularized homografts has better results than with cryopreserved homografts and that results are even comparable to the Ross procedure and mechanical AVR [9]. Kaplan–Meier estimated overall survival was 97.8% and freedom form aortic valve re-operation was 85% at 5 years respectively [9]. Recellularization is more likely to occur, when there are a near-normal anatomic position and blood flow, and avoidance of wrapping procedures and the use of foreign material or tissue glue are recommended to prevent recellularization obstruction [10]. The five decellularized homografts in our cohort, which have been implanted since 2017 had uneventful perioperative course and remain free from re-operation with a short mean follow-up time of 0.2 ± 0.4 years.

The typical limitations, which are imminent to a retrospective study design are present in this study. It is possible that some aspects of surgical as well as postoperative treatment evolvement are not fully accounted for in our comparison by AVR type, though the use of a mechanical or a biological AVR was equally distributed (*p* = 0.337) over the years. Nonetheless, this study offers a long patient-related follow-up time (median follow-up time of 7 years with a maximum follow-up of 32.5 years; 385 patient-years) and on account of a near-complete mortality follow-up (92.7%; 51/55) a patient rather than valve-related outcome analysis, which is an essential aspect regarding reoperation burden over lifetime and late mortality.

## Conclusions

Re-operation was less frequent in the mechanical AVR cohort than in the biological AVR cohort consisting of homografts and bioprosthetic valve replacements. Regarding re-operation rates mechanical valve replacement is favorable, but the risk for thromboembolic and bleeding events was considerable with a composite linearized event rate per valve-year of 3.2%. Longer follow up times of AVR with decellularized homografts must be awaited to compare outcomes.

## Data Availability

The datasets analysed during the current study are available from the corresponding author on reasonable request.

## Abbreviations

AVR: Aortic valve replacement
CI: Confidence interval
ECMO: Extracorporeal membrane oxygenation
HZ: Hazard ratio
INR: International normalized ratio
IQR: Interquartile range
LVAD: Left ventricular assist device
